# MHealth to Improve Measles Immunization in Guinea-Bissau: Study Protocol for a Randomized Controlled Trial

**DOI:** 10.2196/resprot.5968

**Published:** 2016-07-27

**Authors:** Emil Rossing, Henrik Ravn, Celso Soares Pereira Batista, Amabelia Rodrigues

**Affiliations:** ^1^ OPEN, Odense Patient data Explorative Network, Odense University Hospital Department of Clinical Research University of Southern Denmark Odense C Denmark; ^2^ Bandim Health Project Indepth Network Bissau Guinea-Bissau; ^3^ Research Center for Vitamins and Vaccines CVIVA Statens Serum Institut Copenhagen Denmark

**Keywords:** mHealth, eHealth, SMS reminders, voice reminders, Guinea-Bissau, ODK, Africa, RapidSMS, health systems strengthening, randomized controlled trial, measles, immunization

## Abstract

**Background:**

Recent studies have revealed a low measles vaccination (MV) rate in the Republic of Guinea-Bissau (West Africa) that has not increased in accordance with the increasing coverage of other vaccinations. Measles is the deadliest of all childhood rash/fever illnesses and spreads easily, implying that if the vaccination coverage is declining there is a significant risk of new measles outbreaks [27]. Meanwhile, mobile health (mHealth; the use of mobile phones for health interventions) has generated much enthusiasm, and shown potential in improving health service delivery in other contexts.

**Objective:**

The aim of this study is to evaluate the efficiency of mHealth as a tool for improving MV coverage while contributing to the mHealth evidence base.

**Methods:**

This study will take place at three health centers in different regions of Guinea-Bissau. Participants, defined as mothers of the children receiving the MV, will be enrolled when they arrive with their children at the health center to receive the Bacillus Calmette-Guérin vaccination, usually within one month of the child’s birth. Enrolment will continue until a study population of 990 children has been reached. The participants will be randomly assigned to a control arm or one of two intervention arms. Each of the three groups will have 330 participants, distributed equally between health centers. Participants in the first intervention arm will receive a scheduled short message service (SMS) text message reminding them of the MV. Participants in the second intervention arm will receive a voice call in addition to the SMS message, while the control arm will receive no interventions. The MV is scheduled to be administered at 9 months of age. Although the vaccine would still be effective after 12 months, local policy in Guinea-Bissau prevents children aged >12 months from receiving the vaccination, and thus the study will follow-up with participants after the children reach 12 months of age. Children who have not yet received the MV will be offered vaccination by the project group.

**Results:**

The study will analyze the efficiency of the intervention by determining its overall effect on MV coverage and timeliness when children reach 12 months of age. The main analysis will be stratified by intervention group, health center, level of education, ethnic group, and role of the person receiving the text messages (eg, mother, father, other family member). Secondary outcomes include the average number of health center visits (with intention to obtain the MV) required before successful administration.

**Conclusions:**

Despite the rapid proliferation of mHealth projects, only a small number have been evaluated in terms of direct links to health outcomes. This gap in knowledge requires solid evidence on which policy-makers can base decisions. This study aims to produce significant knowledge about mHealth implementation within a Sub-Saharan context while creating data-supported evidence.

**Trial Registration:**

Clinicaltrials.gov: NCT02662595; https://clinicaltrials.gov/ct2/show/NCT02662595 (Archived by WebCite at http://www.webcitation.org/6jH8YiSjY)

## Introduction

### Background

The use of mobile phones as a tool for health interventions (mHealth) has shown significant potential worldwide, and has been used in low income settings. The benefits of such interventions include low start-up costs [1] and the possibility of reaching a great number of people. According to recent reports [2,3], a number of mHealth initiatives have already been conducted around the world. Despite these advances, mHealth has untapped potential, especially in the African region, where the number of such initiatives is the lowest in the world [3]. Although the rapid proliferation of mHealth projects has generated enthusiasm [4], more evidence establishing their efficacy and effectiveness is needed [3,5,6].

Current studies from the International Network for the Demographic Evaluation of Populations and Their Health (INDEPTH) and the Global Alliance for Vaccines and Immunization (GAVI) reveal a low measles vaccination (MV) rate in Guinea-Bissau that has not increased in accordance with the increasing coverage of other vaccinations. In 2014 the United Nations International Children's Emergency Fund (UNICEF) estimated that MV coverage among children 12-23 months of age in Guinea-Bissau was 64% [7], a number that has steadily decreased since 2004 [8].

Meanwhile, other studies have shown that mHealth interventions can be efficient in increasing awareness and demand for health services [9-14]. An important prerequisite for mHealth projects to be successful is access to mobile phones, which has grown rapidly in many developing countries. According to the World Development Indicators provided by The World Bank, it was estimated that in 2013 there were 74 cellular subscriptions per 100 people in Guinea-Bissau (compared to 45 in 2011 and 63 in 2012) [15].

Measles is the deadliest of all childhood rash/fever illnesses and spreads easily, implying that if the MV coverage is declining [16] there is a significant risk of new measles outbreaks [17]. Recent reports from World Health Organization conclude that increased activities are needed to resume progress towards the 2015 Millennium Development Goals of measles control and elimination [18,19]. In addition, recent studies have lead the Strategic Advisory Group of Experts on Immunization to suggest that MV is associated with possible beneficial effects on all-cause mortality [20]. Interventions that enhance the timeliness and coverage of MV are therefore likely to have a great beneficial impact on child health. Thus, we suggest an mHealth intervention to increase MV coverage by sending vaccination reminders by short message service (SMS) texts to mothers before their child’s scheduled MV. While this intervention aims at increasing awareness amongst mothers, it also presents potential benefits regarding the supply of MVs. Current policies in Guinea-Bissau require a minimum number of children to be present before a vial of vaccine can be opened, and since the health centers typically only administer vaccinations one or two days per week, mothers will often the be sent home and told to return a different day. This intervention has the potential to coordinate the vaccination visits and help ensure that enough children are present for the health centers to open a vial of vaccines on a given day, and ensure that mothers arrive on the correct day at the health centers.

Studies from the INDEPTH Network and GAVI have demonstrated that rural regions have particularly low MV coverage, so this intervention will focus on the rural regions of Tombali, Gabu, and Cacheu in the Southern, Eastern, and Northern Guinea-Bissau, respectively.

### Objectives

The primary objective of this intervention is to enable a beneficial impact on child health by improving timeliness and coverage of MVs using simple and cost-efficient methods. Specifically, the study aims to investigate whether a mobile phone targeted reminder system can be used in the context of rural Guinea-Bissau, and what effect this intervention can be expected to have, as well as potential obstacles and barriers to implementation.

## Methods

### Trial Design

**Figure 1:**
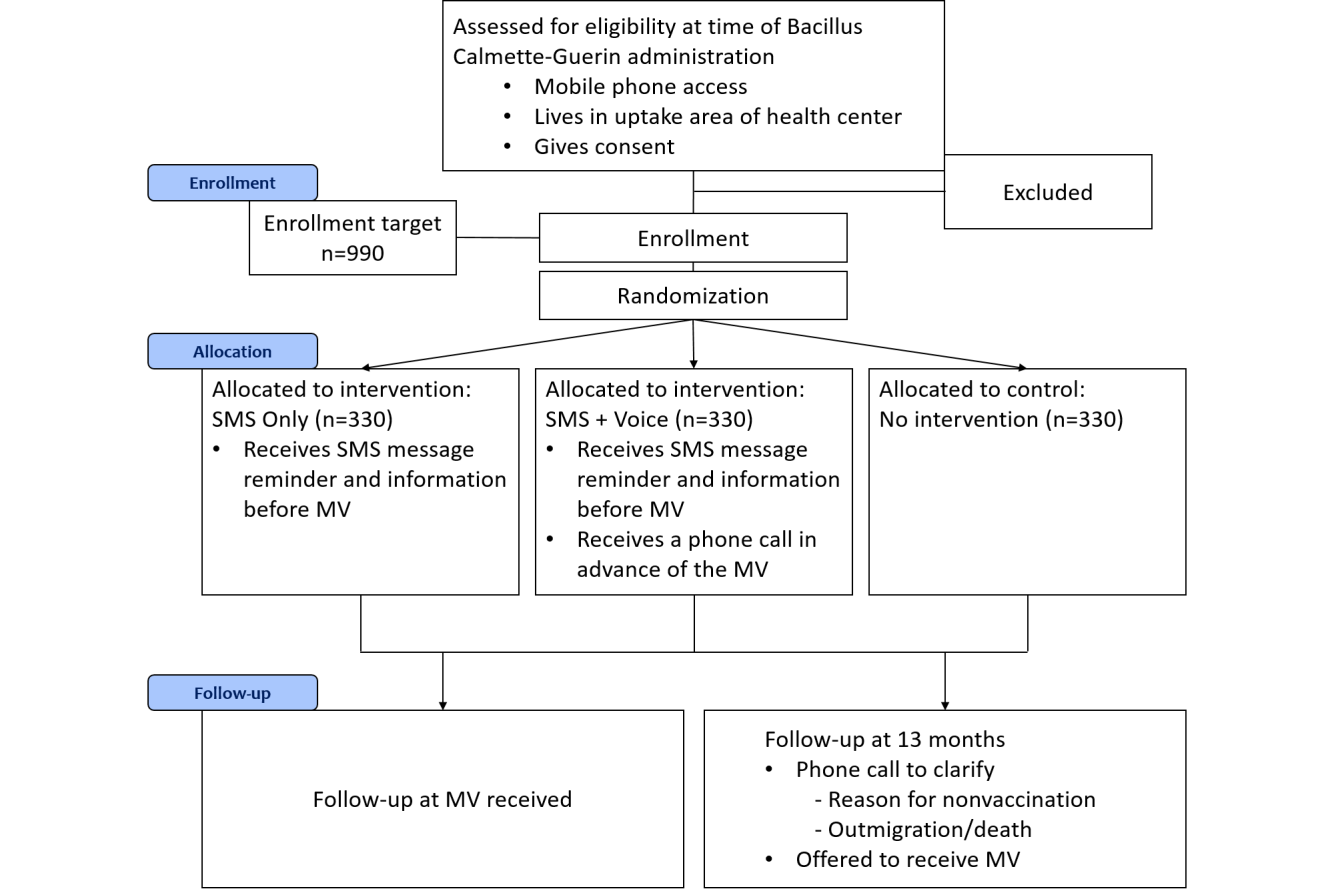
Study flow.

This study is designed as a multi-site, three arm, parallel randomized controlled trial (RCT). Participants will be randomized to either one of two intervention arms (one receiving an SMS reminder, the other receiving the SMS reminder and an additional voice call) or a control arm (no intervention) with a 1:1:1 allocation ratio (Figure 1).

### Informed Consent Forms

Informed consent will be obtained for all study participants before enrollment. The study will be described to the potential participants by trained study workers at the health centers. The project description will be explained in the preferred language of either Portuguese or Creole. After the project has been explained, the potential participant will be asked a number of questions related to the project to ensure adequate understanding, and informed consent forms will be signed.

### Randomization and Blinding

Three randomization sequences (one per health center) were generated using a software algorithm. The randomization sequences are only accessible for the central project group, and will be used when the central project assistant is adding study participants to the scheduling system and delivering phone messages. Participant allocation is concealed to the study assistants at the health facilities, and will only be revealed during the follow-up interview when, and if, the participants come to the health facility to receive MVs.

### Setting and Participants

Study participants, defined as mothers of the children receiving the MV, will be enrolled at one of three selected health facilities when their children receive their first vaccination, usually the Bacillus Calmette-Guérin vaccine, which is administered shortly after birth. The study will be explained to the mothers by the health workers in the local language (Portuguese Creole), and pending their informed consent, the mothers will be enrolled in the study. Enrollment is completed by the health worker, who will take note of the participant’s phone number(s), date of birth, and the baby’s name. A sticker with a unique study identification number will be placed on the child’s vaccination card to indicate that this child is participating in the study.

The inclusion criteria for enrollment in the study are that the participant has access to a cell phone (either her own phone or the phone of a household member or community contact) and that she lives in the uptake area of the health center.

A study assistant will collect information regarding the participant, including age, ethnic group/primary language, and level of education. This information will be entered into a digital questionnaire using an Android device. The data will be successively transferred to a central information technology (IT) system, where a central study assistant will randomize participants and enter the data into the system responsible for scheduling and sending SMS messages, and creating call-lists for the participants scheduled to receive verbal messages.

The enrollment period for this study component will continue until a study population of 990 children (see sample size calculation below) has been reached (total enrollment time is estimated to be approximately 6 months). We will follow up after children reach 12 months of age, since MVs are scheduled to be administered at 9 months of age, and local policy in Guinea-Bissau prevents children aged >12 months from receiving the MV. Thus, this component of the intervention is estimated to last no longer than 18 months.

### Intervention

The intervention being evaluated is a cell phone reminder and coordination system, which seeks to coordinate potential recipients of MV with MV opportunities (ie, days in which MV is administered either at their local health center, or at a relevant outreach day). Participants allocated to intervention groups will receive two SMS text reminders in the local language of Portuguese Creole. The first message will be delivered three days before the suitable MV opportunity, and the second will be sent one day before. If the participant is allocated to also receive a phone call, a project worker will call the supplied phone number two days before the MV opportunity.

SMS messages are *active* in the cell phone network for 24 hours, after which the delivery attempt will be considered failed. If the delivery attempt of a first message fails (ie, three days before MV opportunity), the message will be redelivered. If delivery of the second message fails (ie, one day before MV opportunity), it will not be redelivered due to time rendering it irrelevant. If a phone call fails it will be reattempted two times: once on the same day, and a final attempt on the day before MV opportunity. We will register all failed message delivery attempts and account for these in the subsequent analysis.

If several phone numbers are supplied, SMS texts will be sent to all phone numbers. Voice calls will made to one number, and if the call to the first number is not successful, the second number will be attempted, and so on. Voice calls, like the SMS texts, will be made in the Creole language.

If a study participant does not arrive at the MV opportunity, or if the MV opportunity is cancelled or unavailable for any reason, the study participant will receive a new set of messages/voice calls at the next relevant MV opportunity, assuming the child is still eligible (ie, child is 9-12 months of age). The new set of messages will be delivered regardless of whether earlier delivery attempts for the participant failed.

### Follow-Up

When MVs are given in one of the selected health centers, the participants will be registered in the system as having received the vaccination at the given date. The study assistant will also take note of a set of follow-up questions concerning the participants’ motivation for receiving the vaccination as well as a final question as to whether the participant received and understood the text reminder/voice call. The answers to these questions will eliminate the blinding of within the study, since the participants’ answers will reveal their allocation group. In addition, data regarding price of phone call and time spent per phone call will be collected after each call, and analyzed in terms of cost effectiveness.

A qualitative analysis will be done when children reach 13 months of age, for all participants not registered as having received MVs. The analysis will be based on phone interviews to determine the main reasons for not receiving the vaccine, and it will be registered whether the child has received MV at a different location, moved out of the area, or died. All of these participants will be offered MVs for their children.

A selection of participants from each intervention group (n=10-15), who registered their children as having received the MV, will be visited by a study assistant in order to conduct a face-to-face follow-up interview. These interview will focus on the participants’ perception of the intervention message and the overall experience of the intervention. Data collection will rely on digital questionnaires filled out by project assistants during house visits. Difficulty with logistics (ie, locating and visiting the participants) explain the relatively small number of participants selected for this analysis.

### Location and Duration of the Project

In coordination with the Ministry of Health in Guinea-Bissau, three participating health centers will be selected in the aforementioned regions of rural Guinea-Bissau. The centers will be selected based on the size of the health center (ie, the capacity for enrolling enough study participants during a period of six months), distance to neighboring health centers, mobile cell network coverage, and current level of outreach/other studies.

The first component was carried out in the period from October, 2015 to March, 2016. The second component is scheduled to begin in the first quarter of 2016 and end in the third quarter of 2017 (18 months).

### Development of Reminder Content and Timing

In order to assess the intervention context, and determine the optimal timing and content of the messages to be used in the RCT, a qualitative analysis will be conducted. The analysis will be based on field visits in the rural areas to observe the context for the intervention, including the daily routines for mothers with children aged 6-12 months and their usage of mobile technology. In addition to these observations, a number of semistructured qualitative interviews will be conducted to acquire knowledge about what time of day a message would be preferred, and potential cultural barriers preventing the delivery of messages to household members or neighbors (if the mother does not own a phone).

During the interview, the interviewee will be presented with one of three different versions of the intervention message, and asked questions to evaluate their understanding:

1. A very brief description of measles infection and the reasons for vaccination, inspired by a post card MV reminder intervention that was implementing using the *health belief model* [21].

2. A shorter and more imperative message telling the participant to go to the health center to receive the MV.

3. A verbal message read to the interviewee by the interviewer. The messages will be personalized with the baby’s name and the name of the relevant health center.

All of the messages will be presented in two formats, depending on whether the message is to be delivered directly to the participant’s phone or via the phone of a household member/neighbor. The indirect message variants will be evaluated by presenting them to nearby household members and assessing their understanding and willingness to relay the message.

### Technical Implementation

Building on the efforts of UNICEF, the technical aspects of the project will be based on the open source IT system, RapidSMS. This system is well documented, and has previously been applied successfully in comparative interventions in Kenya and Rwanda [10,22]. The system is customizable and will be adapted to the context of this study. The RapidSMS system will generate reports on SMS texts that failed to be delivered to the recipient (eg, due to cell phone being off for longer periods of time, or a changed phone number). These texts will be delivered again. To enable digital data collection using tablets/phones, an adapted version of Open Data Kit will be used.

To enable capacity building, and thus increase the chances of the system being implemented if the intervention is deemed successful, the technical implementation will be done in close coordination with technical personnel from the national Ministry of Health.

## Results

In order to evaluate mHealth as a viable means of increasing MV coverage and timeliness, an RCT will be conducted. The mHealth intervention will be evaluated using the outcomes in Textbox 1.

**Outcome measures of the randomized control trial.**

- **Primary outcome A:**
  *MV coverage at 12 months of age.* Difference in MV coverage at 12 months of age between the intervention groups and the control group.
- **Secondary outcome B:**
  *Timeliness.* Difference in timeliness of MVs administered, measured as median age of vaccinated children.
- **Secondary outcome C:**
  *Average number of visits* to the health center (with the purpose of receiving MV) needed before MV is successfully administered.
- **Secondary outcome D:**
  *Analysis of context and evaluation* of the different intervention messages.
- **Secondary outcome E:**
  *Cost/benefit analysis of verbal telephone messages versus SMS messages.* The costs in terms of time spent (voice calls), SMS fees, and technical setup will be evaluated against the observed behavior change.
- **Secondary outcome F:**
  *Reasons for nonvaccination.* Qualitative study of the main reasons for noncompliance with the Expanded Program on Immunization, among participants whose children have not received the MV before 13 months of age.
- **Secondary outcome G:**
  *Effect of collecting multiple phone numbers.* Analysis of whether the number of phone numbers collected per participant influences the success rate of the intervention.
- **Secondary outcome H:**
  *Qualitative evaluation* of the participants’ perception of the intervention message and overall experience of the intervention.

### Safety & Ethics

#### Ethical Considerations

One of the key challenges in relation to mHealth is the issue of privacy and data security [23], especially when potentially stigmatizing diseases or substance abuse could be disclosed if family members or others see the text messages, or gain access to the phone used for the intervention [24]. This issue becomes all the more relevant since we have chosen to include mothers, some of whom do not have their own phones, but only have access to shared phones. However, it is our judgment that vaccination reminders do not represent any risk of unwanted disclosure. In addition, a recent study from Argentina concluded that 96% of the study participants (pregnant women attending antenatal care) responded that they would like to receive text messages and phone calls [25]. We have chosen to include participants who do not have their own private phones in an attempt to avoid adding further to the *digital divide* (ie, social inequality due to lack of access to and/or knowledge of information and communication technology).

Another ethical consideration is how to embrace illiteracy/multiple spoken languages without adding to cultural divides or discrimination against certain population segments or ethnic groups. Culture-specific wording of messages is also important, in order to avoid unintended negative effects [26]. At the time of enrollment, trained health workers will describe the study in detail to the potential participants, and assess their understanding in order to obtain their informed consent. All information will be available in Creole (the primary spoken language of Bissau).

To avoid sending SMS reminders to participants who have died or moved out of the study area, or those whose children have died or received the vaccine ahead of schedule, follow-up will be done to the extent possible at the health facilities. It is likely that some of these events will go unnoticed, potentially resulting in inappropriate messages being sent. This problem does not differ from current practices of the Health and Demographic Surveillance System routines, in which families are occasionally asked about deceased members of the household. The possibility of receiving a message regarding a deceased child will furthermore be explained during the informed consent phase.

#### Registration and Ethical Approvals

This project has obtained the approval of the ethical committee of Guinea-Bissau as well as a guiding statement from the ethical body Udlandsudvalget in Denmark. The project has also registered with clinicaltrials.gov (reg.no. NCT02662595).

### Data Management & Statistical Analysis

#### Data Management

The data will be collected using digital questionnaires and stored in a protected database. The data will not be shared with people or organizations outside of the Bandim Health Project, unless proper authorizations are obtained from the ethical committee in Guinea-Bissau. However, the collected data might be made available to an external project group as part of ongoing monitoring and evaluation efforts.

#### Sample Size Calculations

Current statistics indicate MV coverage of approximately 75% at 12 months of age in the rural areas of Guinea-Bissau. Drawing on experiences from other studies implementing SMS reminders [13], we aim for an increase in MV coverage of 10 percentage points. Using a significance level of 5% and 80% power, and a hypothesized increased MV coverage to at least 85% in each of the intervention arms (compared to the control arm), a total of 810 children are needed (270 in each arm). Adjusting for expected loss-to-follow-up between 15-20% (eg, outmigration, death, children being vaccinated elsewhere), which is comparable to other mHealth studies [27], we plan to enroll 990 study participants (330 in each arm).

The current median age of MVs given before 12 months of age in rural Guinea-Bissau is approximately 295 days of age [7]. The age distribution is fairly normal, and in the following power calculation we have used a normal approximation: with 270 children in each arm and an MV coverage of at least 75%, we are able to detect a change of 7 days in median age (using a significance level of 5% and 80% power).

#### Statistical Analysis

The main outcome of MV coverage at 12 months of age (366 days of age) will be calculated as percent who received MV at the health centers, among all children enrolled. Furthermore, coverage will also be calculated based on follow-up interviews when children reach 13 months of age, among study participants whose children did not receive the MV at the health centers. The coverage (percentages) will be compared using Mantel-Haenszel statistics stratified for health center for each of the intervention groups (SMS and voice calls) compared with the control group. The secondary outcome B (Textbox 1) of timeliness of MVs will compare age distributions between vaccinated children, among all children having received MVs before 12 months of age (366 days), using linear regression adjusted for health center. A statistical significance level of 5% will be used. A subanalysis will stratify for study participants having their own private mobile phone versus those using the phone number of another community member. Due to the groups being randomized, we do not expect baseline imbalances. In the case that such trends do occur, we will be unable to rule out other imbalances or unmeasured confounders. The main outcome will therefore be the crude estimate. However, we will investigate the impact of adjusting for unbalanced background factors in sensitivity analyses. The study will be conducted and reported in accordance with Consolidated Standards of Reporting Trials guidelines [28].

## Discussion

### Possible Constraints

External threats to the project, such as strikes in the health sector, shortages of measles vaccine, and other interventions in the same region, will prompt ad-hoc decisions. Problems with vaccine delivery to the vaccination sites will result in the rescheduling of intervention messages (eg, the reminder messages can be rescheduled until vaccine is restocked).

### Dissemination of Results and Publication Policy

Articles are planned for each of the main study components. The main article, concerning the second study component, will contain a statistical analysis linking the effect the mHealth intervention to health outcomes in terms of MV coverage and timeliness. The primary author of the article will be Emil Rossing, with the remaining core members of the project team listed as contributing authors.

A second article will analyze the cost and effect of the mHealth intervention, linking the intervention to vaccination coverage, and add to the evidence base for mHealth interventions. In addition to these articles, the findings of this study will be presented to the National Ministry of Health in Guinea-Bissau, which will also be included as much as possible during the project implementation.

### Limitations and Anticipated Problems

Several problems are anticipated in this study, including technical obstacles such as an unstable cellphone network, and low or no signal in some areas. In order to mitigate these issues, extra attention will be given to *delivery reports* to ensure that the text messages are delivered correctly. In addition, the impact of network-related problems will be covered by the follow-up questionnaire.

The project is also vulnerable to other interventions related to the MVs that are being carried out within the study area, since this could distort the findings of this study. To minimize this risk, the study team will coordinate closely with the National Ministry of Health in Guinea-Bissau.

By only enrolling participants who have access to mobile telephones, there is a risk of potentially adding to social injustice by focusing on a selected demographic with comparably better access to resources, sometimes referred to as *digital divide*. However, we have tried to mitigate this problem by defining *access to a mobile telephone* in the broadest sense possible, by also including participants who will receive the reminder message on the phone of their community’s *health communication agent*. Such agents will be community residents employed by the Ministry of Health as contact points to disseminate information about health events, such as vaccination campaigns. These agents will also help to mitigate the limitation of study participants that do not speak Creole. However, the fact that there are multiple spoken languages in the study area still poses a limitation to the project.

## Abbreviations

GAVI: Global Alliance for Vaccines and Immunization
INDEPTH: International Network for the Demographic Evaluation of Populations and Their Health
IT: information technology
mHealth: mobile health
MV: measles vaccination
RCT: randomized controlled trial
SMS: short message service
UNICEF: United Nations International Children's Emergency Fund
